# Mitigating Bias and Error in Machine Learning to Protect Sports Data

**DOI:** 10.1155/2022/4777010

**Published:** 2022-05-11

**Authors:** Jie Zhang, Jia Li

**Affiliations:** Zhengzhou Preschool Education College, Zhengzhou, Henan 450000, China

## Abstract

One of the essential processes in modern sports is doping control. In recent years, specialized methods of artificial intelligence and large-scale data analysis have been used to make faster and simpler detection of violations of international regulations on the use of banned substances. The smart systems in question depend directly on the quality of the data used, as high-quality data will produce algorithmic approaches of correspondingly high quality and accuracy. It is evident that there are many sources of errors in data collections and intentional algorithmic interventions that may result from cyber-attacks, so end-users of artificial intelligence technologies should be able to know the exact origins of data and analytical methods of these data at an algorithmic level. Given that artificial intelligence systems based on incomplete or discriminatory data can lead to inaccurate results that violate the fundamental rights of athletes, this paper presents an advanced model for mitigating bias and error in machine learning to protect sports data, using convolutional neural network (ConvNet) with high-precise class activation maps (HiPrCAM). It is an innovative neural network interpretability technique, wherewith the addition of Bellman reinforcement learning (BRL) and Broyden–Fletcher–Goldfarb–Shanno (BFGS) optimization; it can produce high-precision maps that deliver high definition, clarity, and the input and output capture when the algorithm makes a prediction. The evaluation of the proposed system uses the Shapley value solution from the cooperative game theory to provide algorithmic performance propositions for each of the produced results, assigning partial responsibility to parts of the architecture based on the impact that the efforts have on the relative success measurement, which it has been preset.

## 1. Introduction

With the commercialization of sport, the lure of a brilliant career with plenty of money and fame is great. Champion-protagonists, whether they are popular team sports or individuals, are idols. The use of substances to increase performance is a well-known practice that concerns the authorities worldwide and those involved in the championship. Doping [1] is related to substances such as anabolic steroids, stimulants, drugs, diuretics, creatine, and many other substances and methods that are very harmful to health and receiving them in large doses for a long time can cause severe problems or even death [2].

An athlete can be tested for doping according to a specific procedure both after a sporting event and without warning during training [3]. Efforts are being made at the national and international level to prevent and reduce the use of doping, which includes, among other things, controls of competitors during nonwarning races [4]. In recent years, specialized methods of artificial intelligence and large-scale data analysis have made it faster and simpler to detect violations of international regulations on banned substances and drugs [5, 6]. The intelligent systems in question depend directly on the quality of the data used, as high-quality data will produce algorithmic approaches of correspondingly high quality and accuracy [7].

There are many errors in data collections and intentional algorithmic interventions that may result from cyber-attacks. Malware can infiltrate a system and change the results of some samples, a process that can easily be proven by repeating the test. However, there are cases where the penetration into the system may involve data alteration or, even worse, the configuration of the artificial intelligence system used to evaluate the samples. Machine learning holds enormous promise for enhancing products, processes, and research. However, computers typically do not explain their predictions, a hurdle to machine learning adoption. Finding patterns and structures in massive amounts of data in an automated manner is a critical component of data science. It is now driving applications in fields as disparate as cybersecurity. However, such a huge positive influence is accompanied by a significant challenge: how can we grasp the decisions proposed by these algorithms to trust them.

The reason is that machine learning techniques were initially designed for stable environments where training and test data come from the same statistical distribution. However, when these models are applied in the real world, the presence of intelligent and adaptive opponents may, depending on the opponent, to some extent violate this statistical hypothesis. By this logic, a malicious opponent can secretly falsify the input data or parameters of the model to exploit specific vulnerabilities of the learning algorithms and endanger the system's security. So, the end-users of artificial intelligence technologies and especially of high importance systems such as antidoping control [8] should be able to know the exact sources of the data and the analytical ways of using and analyzing these data at an algorithmic level [5].

The need for interpretable and explainable machine learning techniques stems from the need to design intelligible machine learning systems, that is, ones that can be comprehended by a human mind, as well as to understand and explain predictions made by opaque models, such as deep neural networks or gradient boosting machines. The interpretability and explainability [9–11] of neural networks are broad. Usually, they have to do with the ability of the algorithm to explain its decisions and whether humans understand the network behavior. If we know the network's input, we can predict and interpret its output. This process is inherent in simple models but practically impossible to achieve in deep neural networks [9, 12]. In these networks, the basic interpretability technique is CAM. The main problem is that the maps are produced from the last convergent level on CNN, which is much less coherent, so the interpretations are provided without sufficient and precise details [13]. This is problematic for many applications, which require a more specific and detailed justification.

With the rising frequency and complexity of methodologies, stakeholders are increasingly concerned about model disadvantages, data-specific biases, and so on. This study aims to design an architecture that will address the problems mentioned above. Based on CAM, we will try to extend them in such a way as to increase their resolution. This is done by adding BRL- and BFGS-type optimization so that the network can produce high-precision maps that render with outstanding clarity and interpretability, the input and output mapping when the algorithm makes a prediction [14]. After motivating the subject generically, we examine the important developments, including the principles that allow us to study transparent vs. opaque models, as well as model-specific or model-agnostic post hoc explainability approaches, from an organizational standpoint. We also give a quick overview of deep learning models before concluding with a discussion of future research areas.

## 2. Related Literature

The literature utilizes the terms interpretability, explainability, and class activation mapping to mitigate the issue of doping that is becoming more sophisticated [15].

Finding appropriate mathematical tools to model deep neural networks' expression ability and training ability and gradually transforming parameter-based deep learning based on empiricism into deep learning based on quantitative guidance of some evaluation indicators is a new topic in artificial intelligence research. The authors of the [16] they study how the neural network search technology in autonomous machine learning can be used as a tool to assist people in furthering their understanding of the “black box” problem of artificial intelligence.

Angelov et al. [9] pinpointed explainability and proposed a solution that addresses the bottlenecks of the traditional deep learning approaches. A deep learning architecture linked reasoning and learning together, which they delivered. It is noniterative, nonparametric, and human-friendly from the user's point of view. Their method outperformed the other techniques in tough classification cases, including deep learning, accuracy, time to train, and an explainable classifier. They aim to continue their research in developing a tree-based architecture, synthetic data generation, and local optimization to improve the proposed deep answerable approach.

Mehrotra et al. [17] stated that when the protected attributes were noisy or missing some or all of the entries, it was also attempted to counteract bias in a selection. Algorithms need to account for real-world noise to avoid bias. There was some thought put into a model of noise in which the protected properties were given a probability. They created a framework for mitigating bias that could satisfy a wide range of fairness requirements with a minimal multiplicative error and a high degree of probability. Their empirical analysis found that their methodology could achieve a high level of fairness on standard measures, even when the probabilistic information regarding protected qualities was skewed, and had a better tradeoff between utility and fairness than several previous methods.

In addition, in this study [18], the authors focus on a popular and commonly used XAI method, layer-wise relevance propagation (LRP). LRP has evolved as a method since its first assertion, and a best practice for using the technique has arisen tacitly, based solely on humanly witnessed data. They also study—and for the first time quantify—the effect of existing best practices on feedforward neural networks in a visual object identification context. The results show that the layer-dependent approach to LRP used in recent literature better depicts the model's reasoning while improving object localization and class discriminability.

Leon [15] concentrated on the Shapley value and created a technique for refining the architecture of algorithms based on it. This game-theoretic solution idea measures the importance of each network piece to accomplishment. The final setting was still a classic layered collection of nodes in their scenario. They demonstrated that the quantity of nodes could be massively reduced while keeping a good, user-defined efficiency by using the Shapley value and a hill-climbing process to finish the fine-tuning. They noted in their findings that more network pieces might be reduced simultaneously, resulting in faster execution times and better outcomes. Furthermore, calculation time was not a problem when employing an estimate of the Shapley value since the user could choose between better precision and longer execution time. Finally, many synapses might be destroyed simultaneously, reducing the number of steps required to complete the operation.

Lundberg et al. [11] did an intriguing study on the developing conflict among model accuracy and interpretability. They proposed Shapley Additive exPlanations, a cohesive approach for analyzing predictions. For each estimate, this system gave a significant value to each feature. It featured the discovery of a new class of additive feature significance measures and empirical models, demonstrating that this class has a single answer with a set of desired qualities. The proposed new strategies critical insights gained through the convergence that outperformed earlier methodologies of computing performance and compatibility with guesswork. The development of speedier model-type-specific estimate techniques with limited information, the integration of work on estimating interaction effects from game theory, and the definition of the additional explanatory classifier are all potential future stages.

Finally, in 2016, Zhou et al. [19] introduced class activation mapping (CAM) for CNNs with globally averaged mixing. They could categorize trained CNNs without utilizing any bounding box annotations because of their method. They were able to show the predicted class scores on every given picture using category activation maps, which highlighted the discriminative object sections discovered by CNN. They tested their strategy on semi-supervised object localization and found that their global average pooling CNNs could execute accurate object localization. They also showed that the CAM localization approach applied to additional vision tasks.

## 3. Methodology

A CAM is an input area that activates a CNN for a particular class [19]. With the map of a class, we can interpret that features of the data set make CNN choose the class to which it belongs. This becomes especially interesting when we produce the CAM of the network that predicts the network, where we see where the network focused when it made its prediction. For a network to create CAM, it must combine a global average pooling (GAP) level at the end of its architecture and a unique fully connected (FC) level [20].

For a given convergent network, let *f*_k_(*x*, *y*) be the activation of neuron *k* of the last convergent level, at the location (*x*, *y*). The next level is a GAP that performs the following operation [21]:

(1) $$\begin{array}{c} F^{k}=\sum_{x,y}f_{k}\left(x,y\right). \end{array}$$

Next, the weighted average of all the neurons is passed to the softmax activation function:

(2) $$\begin{array}{c} z_{c}=\sum_{k}w_{k}^{c}F^{k}, \end{array}$$

where *w*_*k*_^*c*^ is the weight of the neuron *k* for class c and *z*_c_ is the value given by the neuron for this class (that is, the input of softmax). Combining the above relationships, the CAM for class *c* can be produced as [22]

(3) $$\begin{array}{c} S_{c}\left(x,y\right)=\sum_{k}w_{k}^{c}f_{k}\left(x,y\right). \end{array}$$

A more intuitive explanation is that from the last level weight table, which correlates the GAP output with each output class, we isolate the desired class *c*. The weight table column we isolated shows us how each of the GAP outputs affects this class. Each GAP output, however, is nothing more than the average value of the previous level activation map (i.e., the last convergent). In this sense, by summarizing the map at a value, we can see that map affects the input and to what extent. Due to the cohesive network structure, the local input characteristics are retained in the activation maps [23, 24]. Finally, we create the CAM by combining these two pieces of information, namely the activation maps and their relation to class *c*. We do this by taking the sum of all the maps, weighted by the weight of each one.

To view the maps on the original image, it must be converted to have the same consistency. During the last step of the process, the produced map is of very low coherence. It is an ideal solution for the evaluation and, above all, the interpretability of the categorization process. This is due to the inherent feature of CNN that their last level is much lower than the input. We propose a secondary architecture to solve this problem, which aims to create HiPrCAM.

This technique uses BRL and Quasi-Newton-type optimization [15, 25] to produce high-precision maps that deliver input and output when the algorithm predicts outstanding clarity and interpretability. Specifically, in reinforcement learning, the agent receives a representation of the state of the environment and acts, influencing the next state of the environment and receiving a reward. The reward signal is a sequence of real numbers the agent uses to make decisions. In general, the agent's goal is to maximize the sum of the total rewards he receives from the environment in perpetuity and not maximize the immediate reward. This idea is expressed by the reward hypothesis, according to which any goal can be modeled as maximizing the expected value of the sum of a graded reward signal. Since an agent's goal is to select actions to maximize future returns, the value *γ* = 1 in an ongoing job would make it impossible to compare different values of the random variable. In each case, the discount factor *γ* determines the value of the future rewards. A reward at time *t* + *k* contributes to the sum of the returns. Therefore, the discount factor regulates how vital the long-term rewards are to the agent. For *γ* = 0, the process of maximizing the expected return is reduced to selecting the action with the highest immediate reward. For *γ* ⟶ 1, the agent gives more value to the long-term rewards [26]. The way the agent makes decisions is determined by the policy he follows. The policy is defined as a function *π*: *S* ⟶ *p*(*A*), which corresponds to states in probability distributions in the action area, and we consider that it is stationary [27]:

(4) $$\begin{array}{c} \pi \left(a|s\right)=\text{Pr}\left[A_{t}=a|S_{t}=s\right]. \end{array}$$

The status value function is defined as the function *υ*_*π*_: *S* ⟶ *R* that gives the expected return from a state *s*, assuming that the agent selects actions based on a policy *π*:

(5) $$\begin{array}{c} v_{\pi }\left(s\right)=E_{\pi }\left[G_{t}|S_{t}=s\right],\;s\in S. \end{array}$$

Respectively we can define the state-action value function q_π_: *S* × *A* ⟶ *R*, which gives the expected return from a state *s*, assuming that the agent selects action a and then behaves according to the policy *π*:

(6) $$\begin{array}{c} q_{\pi }\left(s,a\right)=E_{\pi }\left[G_{t}|S_{t}=s,A_{t}=a\right],\;s\in S,\;a\in A. \end{array}$$

A fundamental property of value functions is that they can be expressed retrospectively using the observation that [28]:

(7) $$\begin{array}{c} G_{t}=R_{t+1}+\gamma G_{t+1}. \end{array}$$

And the law of total expectation *E* [X] = *E* [E [X |Y]] we get

(8) $$\begin{array}{c} v_{\pi }\left(s\right)=E_{\pi }\left[R_{t+1}+\gamma G_{t+1}|S_{t}=s\right]\\ =E_{\pi }\left[R_{t+1}+\gamma v_{\pi }\left(s^{\prime }\right)|S_{t}=s\right]. \end{array}$$

And, respectively, for the status-action value function:

(9) $$\begin{array}{c} q_{\pi }\left(s,a\right)=E_{\pi }\left[R_{t+1}+\gamma q_{\pi }\left(s^{\prime },a^{\prime }\right)|S_{t}=s,\;A_{t}=a\right]. \end{array}$$

Developing the above function for the possible actions from the state's according to the policy *π* and for its dynamics we have

(10) $$\begin{array}{c} v_{\pi }\left(s\right)=\sum_{a\in A}\pi \left(a|s\right)\sum_{r}\sum_{s^{\prime }\in S}p\left(r,s^{\prime }|s,a\right)\left[r+\gamma v_{\pi }\left(s^{\prime }\right)\right], \end{array}$$

which is the Bellman equation for the condition value function [12].

The proposed methodology uses the Bellman equation to implement a learning system that seeks to learn through direct interaction with the environment. When applied to the value function, the Bellman equation separates it into two parts: the current reward and the discounted future values. Specifically, the Bellman equation with the help of *R*_*s*_^*a*^, *P*_*s*,*s*′_^*a*^ is converted to

(11) $$\begin{array}{c} v_{\pi }\left(s\right)=\underset{a\in A}{\sum }\pi \left(a|s\right)\left[R_{s}^{a}+\gamma \sum_{s^{\prime }\in S}P_{s,s^{\prime }}^{a}v_{\pi }\left(s^{\prime }\right)\right]. \end{array}$$

This equation simplifies the computation of the value function, allowing us to find the best solution of a complex problem by breaking it down into simpler, recursive subproblems and finding their optimal solutions rather than summing over numerous time steps. Assuming that the decision for action an in state's has been made, the equation for possible actions a΄ from state s΄ according to policy *π* and its dynamics becomes

(12) $$\begin{array}{c} q_{\pi }\left(s,a\right)=\sum_{a^{\prime }\in A}\sum_{r}\sum_{s^{\prime }\in S}\pi \left(a|s\right)p\left(r,s^{\prime }|s,a\right)\left[r+\gamma q_{\pi }\left(s^{\prime },a^{\prime }\right)\right]. \end{array}$$

Respectively:

(13) $$\begin{array}{c} q_{\pi }\left(s,a\right)=R_{s}^{a}+\gamma \sum_{s^{\prime }\in S}P_{s,s^{\prime }}^{a}\sum_{a^{\prime }\in A}\pi \left(a^{\prime }|s^{\prime }\right)q_{\pi }\left(s^{\prime },a^{\prime }\right). \end{array}$$

**Figure 1:**
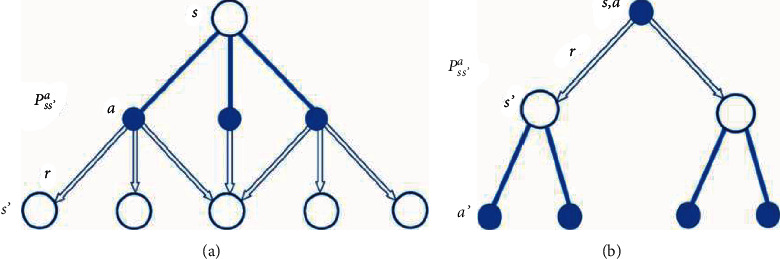
Diagrams for (a) *Vπ* (s) and (b) Q *π* (*s*, *a*) (https://towardsdatascience.com/).

The following two diagrams depicted in Figure 1 explain a standard for identifying the variables and their relationships to facilitate comprehension of the formulation in the suggested approach:

So based on the Bellman equation, we can calculate the value of a state's as the weighted average value according to policy *π* for each pair (*s*, *a*):

(14) $$\begin{array}{c} v_{\pi }\left(s\right)=\sum_{a\in A}\pi \left(a|s\right)q_{\pi }\left(s,a\right). \end{array}$$

Respectively, the value of a state-action pair is equal to the sum of the immediate reward given by the environment and the discounted, weighted according to the dynamics of the environment, average value of each possible next state's [20, 29]:

(15) $$\begin{array}{c} q_{\pi }\left(s,a\right)=R_{s}^{a}+\gamma \sum_{s^{\prime }\in S}P_{s,s^{\prime }}^{a}v_{\pi }\left(s^{\prime }\right). \end{array}$$

The above shows that the specific methodology requires optimization to better deal with non-linear and bad states. The state of a function describes the rate at which the function changes when minor disturbances occur in its input data. Operations that change rapidly with minor changes in data can cause many problems in iterative processes where minor input rounding errors cause significant changes in output [30].

In the proposed smart algorithmic framework, we use an optimization that deals with such objective functions using quasi-Newton type second-order information of the stochastic method. The quasi-Newton method is a class of optimization methods that attempt to address the computationally expensive it is to calculate the Hessian and invert it, especially when dimensions get large. The quasi-Newton approach is used to include multidimensional objective functions. This method imposes additional limitations instead of approximating the second derivative with a finite difference as in the secant technique. However, the standard-issue persists, as each new Hessian must need to be calculated using historical gradient information at each iteration.

So, the BFGS methodology is used, which significantly improves the convergence rates of the technique. Specifically, the iterative formula BFGS for minimizing a twice-continuously generable function *F*: *R*^*d*^ ⟶ *R* is

(16) $$\begin{array}{c} w_{k+1}\leftarrow w_{k}-\alpha_{k}H_{k}\nabla F\left(w_{k}\right). \end{array}$$

**Figure 2:**
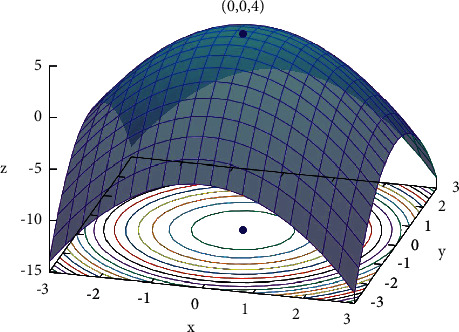
Maximum paraboloid (https://wikipedia.org/).

*H*_*k*_ is a symmetric and positively defined array that approaches the array ∇*F*(*w*_*k*+1_). The difference of the above iterative formula that makes it quasi-Newton is that the sequence {*H*_k_} is updated dynamically when the algorithm is executed and is not just a second-order derivative calculation in each iteration [31, 32]. The maximum paraboloid is presented in Figure 2.

Specifically, the new inverse Essien is given by the difference in the parametric vectors resulting from the iterative process and the difference in the slopes in them [7, 28, 33]:

(17) $$\begin{array}{c} s_{k}≔w_{k+1}-w_{k}\chi \alpha ly_{k}:\\ ≔\nabla F\left(w_{k+1}\right)-\nabla F\left(w_{k}\right). \end{array}$$

The reverse update type of the essential table for the BFGS method is

(18) $$\begin{array}{c} H_{k+1}\leftarrow {\left(I-\frac{y_{k}s_{k}^{T}}{s_{k}^{T}y_{k}}\right)}^{T}H_{k}\left(I-\frac{y_{k}s_{k}^{T}}{s_{k}^{T}y_{k}}\right)+\frac{s_{k}s_{k}^{T}}{s_{k}^{T}y_{k}}. \end{array}$$

The above formula satisfies the quasi-Newton under certain conditions:

(19) $$\begin{array}{c} H_{k+1}y_{k}={\left(I-\frac{y_{k}s_{k}^{T}}{s_{k}^{T}y_{k}}\right)}^{T}H_{k}y_{k}\left(I-\frac{y_{k}s_{k}^{T}}{s_{k}^{T}y_{k}}\right)+\frac{s_{k}s_{k}^{T}}{s_{k}^{T}y_{k}}y_{k}\\ =\left(H_{k}y_{k}-\frac{y_{k}^{T}s_{k}}{s_{k}y_{k}^{T}}H_{k}y_{k}\right)\left(I-\frac{y_{k}s_{k}^{T}}{s_{k}^{T}y_{k}}\right)+\frac{s_{k}s_{k}^{T}}{s_{k}^{T}y_{k}}y_{k}\\ =\left(H_{k}y_{k}-H_{k}y_{k}\right)\left(I-\frac{y_{k}s_{k}^{T}}{s_{k}^{T}y_{k}}\right)+s_{k}=s_{k}\Rightarrow H_{k+1}^{-1}s_{k}=y_{k}. \end{array}$$

The above proves that BFGS has a locally super-linear convergence rate, and this speed is achieved only from first-order information, without the need to solve a linear system, significantly reducing the cost per repetition of the method while ensuring linear convergence.

### 3.1. Method Evaluation

Abnormal Blood Profile Score (ABPS) [28] is used to detect blood doping in sports and was tested using artificial data. As part of the package's ABPS functionality, users must provide the seven hematological marker values for one or more samples. The score or scores will then be calculated and returned. As a single data frame (the basic structure for managing data in R) containing the seven parameters, or by specifying each of the seven variables individually (the standard units are indicated): HCT (hematocrit level, in percent), HGB (the hemoglobin level, in *g*/dL), MCH (the mean corpuscular hemoglobin, in pg), MCHC (the mean corpuscular hemoglobin concentration, in (*g*/dL)), MCV (the Mean corpuscular volume, in fL), RBCs 361 of the 607 cases with fabricated data are expected, and 246 are abnormal.

Initially, a test of the proposed neural network and competing methods was performed to evaluate the categorization ability of the system. The results are presented in Table 1.

The evaluation of the proposed system uses the Shapley value solution from the cooperative game theory, to provide algorithmic performance propositions for each of the produced results, assigning partial responsibility to parts of the architecture based on the impact that the efforts have on the relative success measurement in which they have been preset. Specifically, the Shapley value has been proposed as a cooperation game solution, given as *φi*(*v*) for the *i*th player. It proposes a specific payout for each player from the total winnings from all N players in the game. This share is proportional to how important each player is in the coalition. The foundation of this value was based on four axioms, which are [15, 34–36]:

(1) Symmetry: if *i* and *j* are two players of equal value in a game, i.e., when
  (20) $$\begin{array}{c} v\left(S\cup \left\{i\right\}\right)=v\left(S\cup \left\{j\right\}\right). \end{array}$$
For each coalition *S* of *N*, then *φ*_i_(*v*) = *φ*_j_(*v*).
(2) Cumulative: if two games are combined that have the characteristic equations *v* and *w*, respectively, then the total payout of a player *i* who participates in both games is equal to the payout that he would have separately in the game with characteristic equation *v* plus the payout had separately in the game with distinct equation *w*: *φ*_i_(*v* + *w*) = *φ*_i_(*v*)+*φ*_i_(*w*).
(3) Efficiency: the sum of the payouts of all players is equal to the total payout of the game. The relation describes this condition:
  (21) $$\begin{array}{c} \sum_{i=1}^{n}\phi_{i}\left(v\right)=v\left(N\right). \end{array}$$
(4) Zero player: the value of Shapley *ϕ*_i_(*v*) for each player with zero contribution to the coalition is zero, or otherwise a player's contribution is zero when *υ*(*S Υ*{*i*}) = *υ*(*S*) in a coalition *S*.

The Shapley value satisfies the above four axioms and is given by the relation [37, 38]:

(22) $$\begin{array}{c} \phi_{i}\left(v\right)=\underset{S,i\notin S}{\sum }\frac{n_{s}!\left(n-n_{s}-1\right)!}{n!}\left(v\left(S\cup \left\{i\right\}\right)-v\left(S\right)\right), \end{array}$$

where *n*_S_ is the number of players in the coalition *S*, *n* is the number of players in the game, *v*(*S*) is the value of the characteristic equation for coalition *S*, and *v*(*S* ∪{*i*}) is the value of the characteristic equation for coalition *S* after player *i* joins him.

The factor [*v*(*S Υ*{*i*}− *v*(*S*)] indicates the increase or decrease in the payout of Coalition *S* due to the participation of Player *i* in this coalition. It calculates the extra profit or loss that the involvement will cause to player *i* in an already formed partnership *S*. The factor:

(23) $$\begin{array}{c} \frac{n_{s}!\left(n-n_{s}-1\right)!}{n!}. \end{array}$$

Indicates the probability that player *i* is the (*S*+1) participant in the *S* coalition that already has *n*_s_ players from the *n* participating in the game.

**Figure 3:**
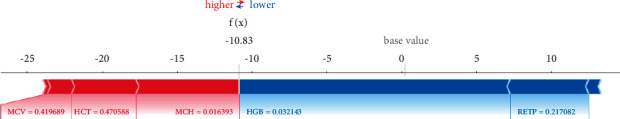
Shapley explanations for the prediction (10 evaluations) of the random sample 156.

**Figure 4:**
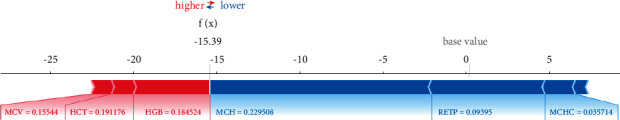
Shapley explanations for the prediction (10 evaluations) of the random sample 309.

**Figure 5:**
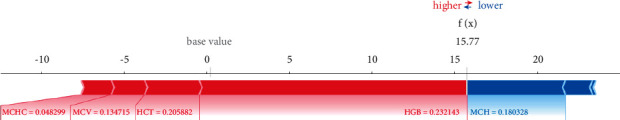
Shapley explanations for the prediction (10 evaluations) of the random sample 567.

The image below uses a selection of a random sample from the data set to represent the typical attribute values. Then ten samples are used to estimate the Shapley values for a given prediction. This task requires 10 × 1 = 10 evaluations of the model.  Figure 3 shows the procedure for sample 156, Figure 4 for sample 309, and Figure 5 for sample 567.

Essentially the Shapley value is the sum of the extra profit (or loss respectively) due to the i-player participation in all possible alliances separately, multiplying the extra profit by the probability that player *i* is the next participant in each association. Thus, the Shapley price gives a unique solution and is monotonous. The greater the player's influence, the greater the payout that he distributes. Shapley values also have universal explanation capabilities, summing the values of a set of samples [34, 35].

**Figure 6:**
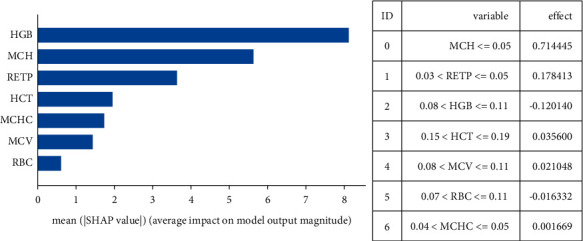
Average impact on model output and effect of each variable.

Extensive research was then conducted to evaluate the values of the variables, how they contribute to the prediction, and to explain each decision of the implemented models using the Shapley values.  Figure 6 shows the classification of the values of the variables used in the bar plot. In contrast, the exact effect value of each is presented in the adjacent table, which shows the period of influence of each variable in the given problem.

**Figure 7:**
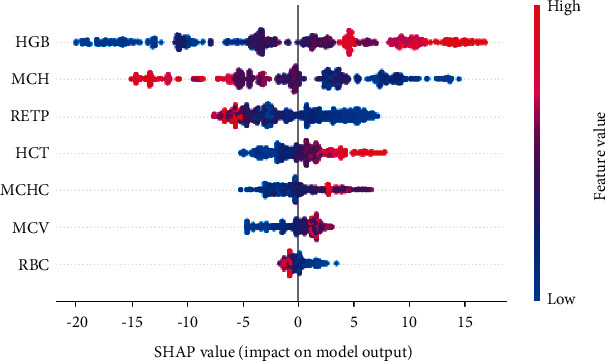
Summary beeswarm plot.

Figure 7 depicts the data set's overall impact concerning each attribute. Each attribute's Shapley values is summed across all samples in the group, and then the details are ranked accordingly. The beeswarm plot provides a concise description of how the top attributes in a data set influence the model's output. The supplied explanation is represented by a single dot on each feature flow in each case. The feature's Shapley value defines the dot's *x* position, and the dots pile-up along with each feature row display density. Color is used to indicate a feature's original value. From top to bottom, the model's most important features are highlighted. Dots represent each feature of the package, and the color of the dot indicates how important it is (blue corresponds to a low value, while red to a high value). The dot's horizontal position on the axis is determined depending on its Shapley value.

We can observe that the HGB feature has the most significant impact on the model predictions. A sample with high Shapley values (red dots) is more likely to be atypical. Because of this, hence the Shapley value has a high positive effect. On the other hand, the Shapley value harms the forecast because it has low values (blue dots). This means that it raises the possibility that the forecast does not come from a standard sample [27, 39].

As it is understood, the proposed model can identify the most critical areas of the entrance and at the same time provide clear explanations for the final decision of the problem. Thus, the information passed to the classifier during the training becomes less and less until we have reached the slightest possible input that does not affect his predictive ability. At the end of the training, the model has already learned to recognize the essential pieces of information provided by the class identifiers.

## 4. Conclusions

In this work, an intelligent framework for protecting sensitive data with explainable artificial intelligence methods has been proposed. Specifically, using an innovative ConvNet assisted by a combined system of an innovative BRL system optimized with the BFGS algorithm, it produces HiPrCAMs, which fully explain and render the input and output mapping with great clarity when the algorithm makes a prediction.

The test of the proposed system was performed on a set of data related to detected in the blood of athletes if there are illegal substances. Respectively, the evaluation of the method was done using Shapley values, which are inspired by the cooperative game theory, to provide algorithmic performance proposals for each of the produced results, assigning partial responsibility to parts of the architecture based on the effect they have on the final decision.

The extension of the proposed system with additional possibilities for recording local and universal variables and their dependence on intermediate representations of the neural network is considered very important to achieve even more accurate and complete knowledge of using the input data.

## Figures and Tables

**Table 1:** Comparison of performance.

ML method	Recall	Precision	f1-score	Accuracy	auc
Proposed nn	92.85	92.80	92.80	92.86	0.9678
mlp	89.80	89.90	89.90	89.91	0.9317
svm	92.90	93.00	92.95	92.99	0.9898
xgboost	93.10	93.15	93.15	93.23	0.9916

## Data Availability

The data used in this study are available from the corresponding author upon request.

## References

[1] Baron D. A., Martin D. M., Abol Magd S. (2007). Doping in sports and its spread to at-risk populations: an international review. *World Psychiatry: Official Journal of the World Psychiatric Association (WPA)*.

[2] Schneider A. J., Friedmann T. (2006). The problem of doping in sports. *Gene Doping in Sports: The Science and Ethics of Genetically Modified Athletes*.

[3] Sharma B. (2022). A Critical Analysis of the Impact of Doping in Sports Domain. *International Journal of Law Management & Humanities*.

[4] Negro M., Marzullo N., Caso F., Calanni L., D’Antona G. (2018). Opinion paper: scientific, philosophical and legal consideration of doping in sports. *European Journal of Applied Physiology*.

[5] Rahman M. R., Bejder J., Bonne T. C. (2022). AI-based Approach for Improving the Detection of Blood Doping in Sports. http://arxiv.org/abs/2203.00001.

[6] Lima G., Muniz-pardos B., Kolliari-turner A. (2021). Anti-doping and other sport integrity challenges during the COVID-19 pandemic. *The Journal of Sports Medicine and Physical Fitness*.

[7] Vamathevan J., Clark D., Czodrowski P. (2019). Applications of machine learning in drug discovery and development. *Nature Reviews Drug Discovery*.

[8] Kelly T., Beharry A., Fedoruk M. (2019). Applying Machine Learning Techniques to Advance Anti-doping. https://www.scholarsresearchlibrary.com/articles/applying-machine-learning-techniques-to-advance-antidoping-18437.html.

[9] Angelov P., Soares E. (2020). Towards explainable deep neural networks (xDNN). *Neural Networks*.

[10] Carletti M., Masiero C., Beghi A., Susto G. A. Explainable machine learning in industry 4.0: evaluating feature importance in anomaly detection to enable root cause analysis.

[11] Lundberg S., Lee S.-I. (Nov. 2017). A Unified Approach to Interpreting Model Predictions. http://arxiv.org/abs/1705.07874.

[12] Gawlikowski J., Tassi C. R. N., Ali M. (Jul. 2021). A Survey of Uncertainty in Deep Neural Networks. http://arxiv.org/abs/2107.03342.

[13] Cano-Berlanga S., Giménez-Gómez J.-M., Vilella C. (2017). Enjoying cooperative games: the R package GameTheory. *Applied Mathematics and Computation*.

[14] Dinar A., Wolf A. (1994). International markets for water and the potential for regional cooperation: economic and political perspectives in the western Middle East. *Economic Development and Cultural Change*.

[15] Leon F. Optimizing neural network topology using Shapley value.

[16] Li S., Gao Y. Using NAS as a tool to explain neural network.

[17] Mehrotra A., Celis L. E. Mitigating bias in set selection with noisy protected attributes.

[18] Kohlbrenner M., Bauer A., Nakajima S., Binder A., Samek W., Lapuschkin S. Towards best practice in explaining neural network decisions with LRP.

[19] Zhou B., Khosla A., Lapedriza A., Oliva A., Torralba A. (2016). Learning Deep Features for Discriminative Localization. https://openaccess.thecvf.com/content_cvpr_2016/html/Zhou_Learning_Deep_Features_CVPR_2016_paper.html.

[20] Dhillon A., Verma G. K. (2020). Convolutional neural network: a review of models, methodologies and applications to object detection. *Progress in Artificial Intelligence*.

[21] Md K., Alam R., Siddique N., Adeli H. (2020). A dynamic ensemble learning algorithm for neural networks. *Neural Computing & Applications*.

[22] Rao Q., Yu B., He K., Feng B. Regularization and iterative initialization of softmax for fast training of convolutional neural networks.

[23] Dai J., Li Y., He K., Sun J. (Jun. 2016). R-FCN: Object Detection via Region-Based Fully Convolutional Networks. http://arxiv.org/abs/1605.06409.

[24] Khan A., Sohail A., Zahoora U., Qureshi A. S. (2020). A survey of the recent architectures of deep `s. *Artificial Intelligence Review*.

[25] Gupta S., Al-Obaidi S., Ferrara L. (2021). Meta-analysis and machine learning models to optimize the efficiency of self-healing capacity of cementitious material. *Materials*.

[26] Xie J., Bai X., Feng D., Gan D. (2009). Peaking cost compensation in northwest China power system. *European Transactions on Electrical Power*.

[27] Ahmadlou M., Adeli H. (2010). Enhanced probabilistic neural network with local decision circles: a robust classifier. *Integrated Computer-Aided Engineering*.

[28] de Adelhart Toorop R., Bazzocchi F., Merlo L., Paris A. (2011). Constraining flavour symmetries at the EW scale I: the A 4 Higgs potential. *Journal of High Energy Physics*.

[29] Thanh Bui L. T., Nguyen Q.-H. (2022). Gradient weighted norm inequalities for very weak solutions of linear parabolic equations with BMO coefficients. *Asymptotic Analysis*.

[30] Zhou B., Khosla A., Lapedriza A., Oliva A., Torralba A. (Dec. 2015). Learning Deep Features for Discriminative Localization. http://arxiv.org/abs/1512.04150.

[31] Garrett A. J. M. (2004). Review: probability theory: the logic of science, by E. T. Jaynes. *Law, Probability and Risk*.

[32] Qian J., Lu J. P., Hui S. L., Ma Y. J., Li D. Y. (2015). Dynamic analysis and CFD numerical simulation on backpressure filling system. *Mathematical Problems in Engineering*.

[33] Ferrari A., Zhao L., Alhoshan W. NLP for requirements engineering: tasks, techniques, tools, and technologies.

[34] Guo B., Hao S., Cao G., Gao H. (2021). Profit distribution of liner alliance based on Shapley value. *Journal of Intelligent and Fuzzy Systems*.

[35] Meng F., Chen X., Zhang Q. (2015). Some uncertain generalized Shapley aggregation operators for multi-attribute group decision making. *Journal of Intelligent and Fuzzy Systems*.

[36] Demertzis K., Tsiknas K., Takezis D., Skianis C., Iliadis L. (2021). Darknet Traffic Big-Data Analysis and Network Management to Real-Time Automating the Malicious Intent Detection Process by a Weight Agnostic Neural Networks Framework.

[37] Petrosyan L., Sedakov A., Sun H., Xu G. (2016). Time consistency of the interval Shapley-like value in dynamic games. *Journal of Intelligent and Fuzzy Systems*.

[38] Lipovetsky S., Conklin W. M. (2010). Meaningful regression analysis in adjusted coefficients Shapley value model. *Model Assisted Statistics and Applications*.

[39] Salasar L. E. B., Leite J. G., Louzada F. (2016). Likelihood-based inference for population size in a capture–recapture experiment with varying probabilities from occasion to occasion. *Braz. J. Probab. Stat., *.

